# Using formative process evaluation to improve program implementation and accessibility of competitive group-based physical activity in the TEAM-PA trial

**DOI:** 10.1186/s12966-024-01635-1

**Published:** 2024-08-13

**Authors:** Allison M. Sweeney, Dawn K. Wilson, Nicole Zarrett, Timothy Simmons, Makayla Mansfield, Lindsay Decker

**Affiliations:** 1 Department of Biobehavioral Health and Nursing Science, College of Nursing, University of South Carolina, 1601 Greene Street, Columbia, SC 29201 USA; 2 Department of Psychology, University of South Carolina, Columbia, SC 29201 USA

**Keywords:** Process evaluation, Physical activity, Group-based interventions, African American women

## Abstract

**Background:**

This study demonstrates how formative process evaluation was used to assess implementation and improve dose and fidelity in the Together Everyone Achieves More Physical Activity (TEAM-PA) randomized controlled trial. TEAM-PA uses a randomized group cohort design to evaluate the efficacy of a group-based intervention for increasing physical activity among African American women.

**Methods:**

Intervention groups met for 10 weeks and were co-led by female African American facilitators, with intervention sessions consisting of group feedback, a health curriculum, group-based physical activity games, and group-based goal-setting. Drawing from a multi-theoretical framework, the intervention targeted social affiliation using collaborative and competitive group strategies, including essential elements focused on group-based behavioral skills, peer-to-peer positive communication, collectivism, optimal challenge, social facilitation, and peer to peer challenges. Formative process evaluation was used to monitor reach, dose, and fidelity, and implement feedback and solutions.

**Results:**

Across two cohorts, four groups (*n* = 54) were randomized to the TEAM-PA intervention. On average 84.8% of participants attended each week, which exceeded the a priori criteria. Results from the systematic observations indicated that on average 93% of the dose items were completed in each session and adequate levels of fidelity were achieved at both the facilitator and group-levels. Participants were compliant with wearing the FitBits (6.73 ± 0.42 days/week) and most participants successfully contributed to meeting the group-based goals. The use of open-ended items also revealed the need for additional modifications to the group-based PA games, including allowing for individuals to take breaks, incorporating a broader range of exercises, minimizing activities that required bending/reaching down without assistance, and providing facilitators with additional training for implementing the games. Initial evidence suggests that these changes were successful in increasing participants’ comprehension of the games from Cohort 1 (*M* = 1.83, *SD* = 0.71) to Cohort 2 (*M* = 3.33, *SD* = 0.69).

**Conclusion:**

Findings from this study demonstrated high levels of reach, dose, and fidelity, while also highlighting strategies for implementing competitive group-based PA games that are accessible across physical fitness levels. Formative process evaluation, including open-ended items and collaborative brainstorming, holds tremendous potential for improving future interventions.

**Trial registration:**

This study was registered on Clinicaltrials.gov (# NCT05519696) on August 22, 2022 prior to the enrollment of the first participant on September 12, 2022 (https://clinicaltrials.gov/study/NCT05519696?term=NCT05519696&rank=1).

## Background

African American women experience a variety of social and structural barriers to physical activity (PA) [1–3], which contribute to high levels of physical inactivity, elevated levels of cardiovascular disease, and pre-mature death [4–6]. Group-based PA interventions may be especially appealing and culturally-relevant to African American women, given that African American women often report unique interpersonal barriers to PA (e.g., low social support, lack of a partner, caregiver responsibilities) [1, 2] and view social connectedness and collectivism as core cultural values [7–9]. Group-based interventions are theorized to be an effective approach for increasing PA due to a variety of interacting processes related to group dynamics, interpersonal factors, and individual-level mechanisms of change [10, 11]. Despite the potential usefulness of group-based approaches, systematic reviews of PA interventions for African American women have yielded mixed efficacy results [12–15], which may be due, in part, to challenges with program implementation. Although there has been a growing interest in recent years in using process evaluation as a key component in evaluating PA interventions [16], these frameworks remain underutilized, as evidenced by a recent systematic review, which found that less than half of PA interventions included a process evaluation framework [17].

Process evaluation, which involves careful monitoring of program delivery and implementation, is a critical tool for maximizing program implementation and treatment outcomes, as well as understanding when interventions are effective or ineffective [18–20]. Process evaluation is often used for summative purposes post-implementation to document whether the intervention was implemented as planned, inform the interpretation of results, and identify implementation components related to treatment outcomes [21–23]. Importantly, formative process evaluation can also be used throughout the delivery of an intervention to allow for ongoing monitoring of program implementation and identifying when adjustments are needed [24–26]. The purpose of the present article is to demonstrate how formative process evaluation is being used in the ‘Together Everyone Achieves More Physical Activity’ (TEAM-PA) trial, which tests the efficacy of a novel group-based social affiliation intervention for increasing total daily PA among insufficiently active African American women using a community-based randomized design.

A comprehensive formative process evaluation approach allows researchers to monitor various implementation components including dose (extent to which program content is delivered and received), fidelity (extent to which the intervention matches the theoretical essential elements and is implemented as planned), and reach (proportion of intended audience receiving the intervention) [20]. Past group-based interventions have also integrated a multi-level approach to distinguish between facilitator-level fidelity (extent to which the facilitators implement the intervention essential elements as intended) and group-level fidelity (extent to which the group climate reflects the intervention essential elements as intended) [24]. By assessing these components in the TEAM-PA intervention, formative process evaluation can help to ensure adherence to the originally designed intervention (e.g., theoretical essential elements), consistency across intervention sites, and reduce the likelihood of unintended ‘protocol drift’ across time. Given that process evaluation frameworks remain underutilized [17], studies are needed that demonstrate how formative process evaluation can be used to monitor and improve program implementation and maximize treatment outcomes, especially PA interventions targeted towards African American women, which have historically yielded mixed efficacy findings.

The TEAM-PA intervention aims to promote social affiliation among group members and is based on essential elements derived from Social Cognitive Theory (SCT) [27, 28], Self-Determination Theory (SDT) [29], and Group Dynamics theory (GDT) [30, 31], and draws from an Afrocentric Worldview [8] by focusing on collectivism. We propose that social affiliation may be especially relevant to African American women because collectivism (belief in the importance of promoting the group over the individual) is a central component of an Afro-Centric worldview [7, 8, 32]. Past studies have found that collectivism is positively associated with other protective cultural factors, including central-internalized Black racial identity and Black cultural strength [9, 33]. While there is likely variability in the extent to which African American women view collectivism as a core cultural value, a broader desire for social affiliation may arise from the heightened discrimination experienced by this group, due to the interaction of race, gender, and other social identities [34].

The TEAM-PA intervention targets social affiliation using both collaborative and competitive group strategies, including essential elements that focus on group-based behavioral skills, peer to peer positive communication, collectivism, optimal challenge, social facilitation, and peer to peer challenges (see Table 1). While past studies have often focused on individual-level behavioral skills (e.g., goal-setting, self-monitoring) [35, 36], the TEAM-PA intervention targets collaborative group-based behavioral skills, where groups work together to develop shared group-based PA goals, receive group feedback, and problem-solve with support from peers, which we propose may be more effective for building social affiliation and increasing PA. Furthermore, while past interventions with African American women have often focused on non-competitive approaches to delivering group PA sessions (e.g., walking, aerobics, dance) [12, 37, 38], the TEAM-PA intervention includes a novel focus on intragroup competition, including competitive group-based PA games, which involve competing in small teams to complete different types of activities, such as relays and calisthenic challenges. Although past research suggests that individual-level competition may have a detrimental effect on motivation [39], intragroup competition has been associated with several positive group outcomes, including enhanced performance and group cohesion [40, 41], especially among women [42].

**Table 1:** TEAM-PA theories and essential elements

Theory	Essential elements	Description of program elements
SCT, GDT	Group-based behavioral skills	Participants share anticipated or actual physical activity barriers and brainstorm problem-solving strategies as a group.
		Participants select a weekly collective group-based PA goal, which they track with their Fitbits.
SDT, GDT	Peer to peer positive communication	During the group sessions, the facilitators reinforce positive interactions between group members (e.g., sharing ideas, words of encouragement).
		Participants are encouraged to support each other throughout the week by posting to the private group or sending messages through the Fitbit mobile app. A weekly “team captain” is selected to post an update in the private group and encourage the group.
Afro-Centric Worldview	Collectivism	Facilitators emphasize the importance of group performance (e.g., group goals, total distance covered by the group in steps)
		PA curriculum integrates social-cultural topics related to collectivism
SDT	Optimal Challenge	Participants complete competitive group activities at each session, which balance novelty and competency.
SCT, GDT	Social Facilitation	Participants monitor their personal progress and the group’s progress in meeting the weekly physical activity goal through the Fitbit mobile app, including a mid-week text reminder from the facilitators to check their progress on the FitBit mobile app.
GDT	Peer to peer challenges	Participants are encouraged to use the leaderboard feature on the Fitbit mobile app to engage in peer to peer challenges (to further the group’s overall performance), including a mid-week text from the facilitators to highlight the group’s mid-week performance.

*Note* SCT = Social Cognitive Theory; SDT = Self-Determination Theory; GDT = Group Dynamics Theory. This table was originally published in Contemporary Clinical Trials [46]

Support for this approach comes from a series of qualitative and pilot studies which included community member input and demonstrated the feasibility, acceptability, and preliminary impact of integrating a team-based approach targeting collaboration and intragroup competition among African American women [43–45]. Specifically, we observed high levels of feasibility and acceptability for the collaborative and competitive components, and a clinically meaningful pre-post increase in minutes/day of total PA [44, 45]. While these preliminary studies provide an important example of how to address social affiliation in a group-based PA program for African American women, this initial pilot work was completed in collaboration with a single community site. The current study expands on this work by adding additional formative work around expanding this program of research from the pilot phase to a randomized controlled trial implemented with multiple community sites, the expansion of the competitive group-based PA games, and an added focus on using cultural adaptations to address collectivism.

Implementation of a multi-theoretical framework can be challenging [19, 20], especially across multiple community sites and groups, with group members varying in their PA interests and levels of physical fitness and mobility. Thus, the purpose of the present study is to demonstrate how a multi-component, theory-based process evaluation approach was used to assess implementation and provide formative feedback during years one and two of a group-based social affiliation PA intervention for African American women. Using systematic observations, attendance tracking, and engagement data from FitBits, formative process evaluation was used to evaluate reach, dose, fidelity, and provide timely, corrective feedback and solutions to implementation barriers.

## Methods

### Participants

Participants are recruited to participate in one of two group-based PA programs (TEAM-PA intervention or a standard group-delivered comparison program). To be eligible, participants were required to: (1) be ≥ 18 years old; (2) self-identify as an African American or Black female; and (3) engage in < 60 min of self-reported MVPA per week for the last three months. Exclusion criteria included: (1) having a cardiovascular or orthopedic condition that would limit PA; (2) inability to walk without a walker/cane; (3) pregnancy; or (4) uncontrolled blood pressure (systolic > 180 mmHg/diastolic > 110 mmHg).

### Study design

The TEAM-PA trial is a randomized group cohort design, with each cohort consisting of 3–4 groups of approximately 10–15 participants per group [46]. Recruitment and program implementation are supported by strong community partnerships with local organizations that provide family and community services (e.g., afterschool programs, recreation activities for adults and children). Within these organizations, we identify community sites (e.g., recreation centers) serving a high percentage of African American residents (*≥* 70%), which serve as hubs for delivering the two group-based PA programs. To reduce contamination bias, only one group is delivered at each community site at a time, implementation of the next group is spaced by at least 3 months, and all community sites are at least 15 miles from each other. The trial is being implemented in South Carolina. All community sites are in metropolitan areas (as defined by Core Based Statistical Areas). The full protocol for the trial has been published previously [46].

### Recruitment

Participants first complete a phone screener, including the short-version of the International Physical Activity Questionnaire [47] and the Physical Activity Readiness Questionnaire (2022 Version) [48]. If participants report a history of cardiovascular disease, high blood pressure, orthopedic issues, or other conditions that would limit PA, medical approval from their primary care provider is required. Eligible participants are then invited to a group orientation session, which includes additional information about the study and research team, opportunities for asking questions, a blood pressure assessment, and completion of informed consent. Participants then complete a 2-week run-in period prior to randomization to collect all baseline measures. After run-in, groups are randomized to the intervention or comparison program using a computer-generated randomized algorithm. This paper reports on TEAM-PA intervention groups in Cohorts 1 and 2 of this ongoing trial. Participants (*N* = 54) were between ages 27 and 76, with an average age of 53.48 ± 14.49 years. Approximately 42.6% were married and 31.5% had at least one child living at home. The median annual household income was $40,000 to $54,999, and 61.1% had a college degree or greater. At baseline, most participants had a BMI in the obese range, with an average BMI of 34.84 ± 8.05.

### Overview of the TEAM-PA intervention

The TEAM-PA intervention aims to promote a positive group climate and is based on essential elements derived from Social Cognitive Theory (SCT) [27, 28], Self-Determination Theory (SDT) [29], and Group Dynamics theory (GDT) [30, 31], and draws from an Afrocentric Worldview [8] by focusing on collectivism. The essential elements informed the development of the TEAM-PA intervention (e.g., intervention curriculum, methods, and activities), guided staff training (e.g., weekly intervention facilitator guides), and defined dose and fidelity for the TEAM-PA intervention implementation.

Table 1 provides the theoretically-based essential elements for the TEAM-PA intervention. Drawing from a multi-theoretical framework, the TEAM-PA intervention targets three major program components: (1) collaborative skills and support, including the use of group-based behavioral skills (shared goal-setting and group problem-solving) and peer-to-peer positive communication; (2) friendly intragroup competition (including opportunities for peer-to-peer challenges, social facilitation, and optimal challenge); and (3) a collectivism focus, including emphasizing the group’s PA progress (vs. individuals) and discussion of relevant cultural topics.

The TEAM-PA intervention is delivered by two trained facilitators at community sites. Groups meet weekly for 12 weeks (including the 2-week run-in period) for two hours in the evening.

After the run-in period, participants receive a Fitbit (Inspire model) to track their PA and instructions for using the Fitbit mobile app. Participants are connected as “friends” with their group members on the app and a private group is set up by the research team to facilitate conversations among group members. The intervention group sessions include four major components: group feedback and problem-solving (15 min); delivery of a discussion-based health curriculum (with cultural topics related to collectivism) (30 min); intragroup competitive PA session (30 min); and group-based behavioral skills training (e.g., collective PA goal-setting). Outside of the in-person sessions, participants are encouraged to track their daily PA using the FitBits and use the FitBit mobile app to monitor their team’s progress towards achieving their weekly team-based goal, engage in friendly competition via the app’s leaderboard, and communicate with their team members in the private group. For a detailed description of the intervention approach see the protocol paper [46].

### TEAM-PA intragroup competitive PA sessions

Based on previous qualitative and pilot work by the research team [43–45], the TEAM-PA intervention integrates opportunities for peer-to-peer challenges and group competition. Each week, participants complete 30 min of PA during the group sessions, including a warm-up, a competitive group activity, and a cooldown implemented by the trained facilitators. Guided by SDT and GDT, the goal of these intragroup competitive PA sessions is to provide participants with opportunities to engage in an optimal challenge (balancing competency and novelty) and friendly group competition, which is theorized to be important for promoting group cohesion. For the competitive activities, groups are divided into small teams for relay-based games, calisthenics challenges, or team-based chair exercise challenges, adapted from another community-based trial [49].

### TEAM-PA intervention training

African American female facilitators are trained to co-deliver the programs. Facilitators receive facilitator guides for each group session that outline key content, cultural topics, behavioral skills, and the TEAM-PA intragroup competitive activity. All facilitators complete extensive training, including behavioral skills related to PA through didactic and role-play components. Training targets motivational interviewing skills [50] (e.g., reflective listening, descriptive praise, “push” vs. “pull” language), techniques for promoting a positive social environment, how to target key behavioral skills in group sessions, CPR/first aid, and cultural competency approaches.

### TEAM-PA process evaluation methods

Process evaluation methods were guided by the essential elements framework that defined dose and fidelity of the TEAM-PA intervention. This paper focuses on process evaluation related to reach (proportion of participants who received the intervention as intended), fidelity (extent to which the intervention adheres to the theoretical essential elements as planned), and dose delivered (completeness of all components) in the TEAM-PA intervention [20]. Although our process evaluation approach was both formative and summative, the present paper focuses on the formative process evaluation results from Cohorts 1 and 2, which were used to identify implementation strengths and areas for improvement. In the results, we describe key lessons learned from the formative process evaluation and steps taken to further improve program implementation. For an overview of the process evaluation approach, please see Table 2.

**Table 2:** Overview of the process evaluation approach

Process Component	Method	A priori goal
Reach	*Attendance tracking*: participants sign-in and sign-out at each group session to document the number of participants in attendance each week *Makeup sessions*: if a participant misses a group session, they are contacted within 48 h to schedule a makeup session by phone	Average > = 75% of participants in attendance each week
Dose	*Systematic observational checklist*: a series of yes/no items are used to document whether key program elements were delivered; completed by an independent staff member trained in the intervention	All program component to be delivered > = 75% of the time
Fidelity	*Systematic observational checklist*: a series of items on a 4-point scale (1 = none, 2 = some, 3 = most, 4 = all) are used to document whether the intervention essential elements were delivered as planned; completed by an independent staff member trained in the intervention	Average > 3 on all sub-sections, which may be divided by intervention essential elements and/or group vs. facilitator-levels
Adherence	*FitBit tracking*: participants’ weekly steps and frequency of meeting the team-based goals are tracked to document adherence with using the FitBits as intended	Average > = 5 days/week of wearing the FitBit (defined as > 0 steps/day)

### Attendance tracking

Each week, participants are asked to sign-in and sign-out when attending the group sessions, which is used to track attendance and program reach. The a priori goal for the TEAM-PA trial is to average *≥* 75% of group members in attendance per group each week. If a participant misses a session, facilitators aim to schedule a makeup call with participants prior to the next group session, which has been found to be an important strategy in past group-based trials for sustaining engagement and reducing attrition [51]. Attendance rates are calculated both with and without the inclusion of makeups for comparison.

### Systematic observations

As shown in Table 3, a systematic observational checklist is completed by independent process evaluation staff, which is used to assess fidelity to the essential elements and dose delivered. Based on a previous group-based trial [24, 51], the systematic observation approach is multi-level and focuses on behaviors and interactions between both the facilitators and group members. Independent staff members undergo the same training as intervention facilitators, and complete a certification process, which involves listening to recordings of example sessions, completing practice evaluations, and achieving high interrater reliability (*r* ≥ .80). Evaluators receive copies of the weekly facilitator guides and complete a systematic observation at each group session.

**Table 3:** TEAM-PA intervention fidelity and dose items

Process Component	Example Items	
**Fidelity - Facilitator Level**		
Behavioral skills	Facilitator(s) aid participants in identifying barriers towards skill development and goal attainment	
Positive communication skills	Facilitator(s) use open-ended question to elicit reflections and input from participants	
Social Support	Facilitator(s) acknowledge and reinforce positive interactions between participants	
Session Content	Facilitator(s) covered key content as outlined in the facilitator’s guide	
**Fidelity - Group Level**		
Behavioral skills	Participants help one another identify strategies for overcoming barriers to PA goals.	
Positive communication skills	Participants engage in reciprocal communication with one another	
Group climate	Participants share personal stories related to working on PA goals	
Group climate during PA session	Participants display signs of excitement or fun (cheering, clapping, high fives)	
**Dose**		
Arrival/Set Up	• Session objectives reviewed with participants • Snack and water are offered • Ground rules are displayed	
Health Curriculum	• [Behavioral Skill*]* discussed during session as highlighted in the facilitators guide. • [Collectivism Social-Cultural Topic] discussed during session as highlighted in the facilitators guide.	
PA Session	Most (≥ 75%) of the participants engage in *≥* 30 min of PA during physical activity session	
Goal-Setting/Problem-Solving	Most (> 75%) of participants engage in group feedback (e.g., provide comments, ask questions)	

Items related to dose are answered on a binary scale (0 = No, 1 = Yes), with each item assessing a specific program component. The observational checklist includes a total of 19 dose items, which are organized into sections related to arrival/setup, session content, the PA session, and group behavioral skills/goal-setting. The a priori goal for dose is for all program component to be delivered *≥* 75% of the time. Items related to fidelity are answered on a 4-point scale (1 = none, 2 = some, 3 = most, 4 = all). The observational checklist includes a total of 57 fidelity items, which are organized into sub-sections related to the facilitator (group feedback, social support, communication skills, session content, collectivism topic, behavioral skills, goal-setting) and group levels (communication skills, behavioral skills, and group climate during the PA session). The a priori goal for fidelity is to average *≥* 3 at both the facilitator and group levels for each of these sub-sections. Benchmarks for dose and fidelity are based on previous group-based intervention [24, 51].

Beginning in Cohort 2, additional items were added to the observational checklist to identify potential implementation challenges related to the PA intragroup competitive games, including whether participants engaged in at least 50% of the game, whether one or more participants needed breaks during the game (e.g., to catch their breath, sit down), challenges to implementing the games, and the use of adaptations to the games.

### FitBit tracking

To monitor adherence with using the FitBits and contributing to the collective team-based goals, Fitabase (Small Steps LLC) is used to compile participants PA data during the intervention period. Compliance is tracked by calculating the number of days/week with > 0 steps. The a priori goal was for participants to average > = 5 days/week of FitBit wear. We also evaluated the number of times individuals successfully met or exceeded the shared group-based PA goals.

## Results

### Reach

Across Cohorts 1 and 2, a total of four groups (*n* = 54) were randomized to receive the TEAM-PA intervention. Results of program reach are shown in Table 4. Overall, on average 84.8% of participants were in attendance each week and attended an average of 8.6 sessions (out of 10). With the inclusion of makeup sessions, reach was further improved, with an average of 97% of participants in attendance each week, and an average of 9.5 sessions completed.

**Table 4:** Attendance in the TEAM-PA intervention

	Cohort 1	Cohort 2	Total
*N*	27	27	54
Make-Up not included			
Average participants per session (%)	86.80%	82.90%	84.80%
Average number of sessions attended (*M*, *SD*)	8.8 (1.1)	8.4 (1.5)	8.6 (1.3)
Make-Up included			
Average participants per session (%)	97.00%	97.00%	97.00%
Average number of sessions attended	9.5 (0.7)	9.5 (0.8)	9.5 (0.7)

### Dose delivered

Results from the systematic observations indicated that on average 93% of the dose items were completed in each session, which exceeded our a priori goal of *≥* 75%. Only one item fell below our a priori goal of *≥* 75%, which was “starting the session on time” (*M* = 69%). We observed an improvement in implementation of a few items, including an improvement in explaining the weekly take home challenge (Cohort 1: *M* = 88%; Cohort 2: *M* = 100%) and action plans developed and shared by participants (Cohort 1: *M* = 78%; Cohort 2: *M* = 100%). Overall, dose was high, with several items averaging 100% across both Cohorts, including “group feedback delivered”, “Most (> 75%) key content delivered”, “behavioral skills covered”, and “groups worked together to set a team-based goal”.

### Fidelity

Results of facilitator and group-level fidelity are presented in Table 5 and reveal adequate overall facilitator delivery of the essential elements (*M* = 3.77, *SD* = 0.22). Ratings were highest for session content (*M* = 3.99, *SD* = 0.08), behavioral skills (*M* = 3.91, *SD* = 0.27), and collectivism topic (*M* = 3.86, *SD* = 0.53), and slightly lower for social support (*M* = 3.56, *SD* = 0.40) and facilitator delivery of group feedback (*M* = 3.38, *SD* = 0.40). Group-level fidelity was also adequate (*M* = 3.46, *SD* = 0.39), but somewhat lower than facilitator-level fidelity. In terms of group-level behavioral skills, there was a small decrease in fidelity between Cohort 1 (*M* = 3.69, *SD* = 0.48) and Cohort 2 (*M* = 3.15, *SD* = 0.58), but the a priori goal of averaging *≥* 3 was still met.

**Table 5:** Fidelity of the TEAM-PA intervention implementation, *M* (*SD*)

	Cohort 1	Cohort 2	Total
**Facilitator Level**			
Group feedback	3.41 (0.43)	3.34 (0.38)	3.38 (0.40)
Social Support	3.5 (0.68)	3.62 (0.40)	3.56 (0.40)
Communication Skills	3.78 (0.28)	3.89 (0.11)	3.83 (0.21)
Session Content	3.97 (0.12)	4 (0.0)	3.99 (0.08)
Collectivism Topic	3.72 (0.73)	4 (0.0)	3.86 (0.53)
Behavioral Skills	3.82 (0.36)	4 (0.0)	3.91 (0.27)
Goal-Setting	3.64 (0.40)	3.96 (0.13)	3.8 (0.33)
Overall average facilitator level fidelity	3.69 (0.28)	3.85 (0.11)	3.77 (0.22)
**Group Level**			
Communication Skills	3.53 (0.38)	3.64 (0.16)	3.59 (0.29)
Behavioral Skills	3.69 (0.48)	3.15 (0.58)	3.42 (0.59)
Group Climate during Physical Activity	3.74 (0.44)	3.41 (0.66)	3.57 (0.22)
Overall average group-level fidelity	3.58 (0.39)	3.34 (0.36)	3.46 (0.39)

*Note* Items range from 1 (None) to 4 (All)

### Additional evaluation of the TEAM-PA games and lessons learned

Importantly, the systematic observational checklist revealed that the group-climate during the group-based PA games, which includes behaviors like clapping, cheering, and high-fives, was adequate (*M* = 3.57, *SD* = 0.22). However, in Cohort 2, additional items were added to the systematic observation checklists to provide a more detailed assessment of the implementation of the games and document potential implementation barriers. We found that most of the games (94%) were implemented as planned with all participants (100%) engaging in at least 50% of the game. However, we observed that there were some sessions where one or more participants needed a break between rounds (e.g., to catch their breath or sit down). Additionally, the use of open-ended items on the systematic observation checklist helped the research team to identify key challenges related to implementing some of the games. Solutions were generated through brainstorming sessions with the intervention facilitators and eliciting feedback from participants, and then implemented using an iterative process during Cohort 2.

First, we observed that there was a need to make the games more accessible for all levels of fitness and/or mobility. Solutions to this challenge included: (1) allowing participants to “tag in” a facilitator if they need a short break to play in their place; (2) including a variety of options for different types of body weight exercises (e.g., options to avoid jumping/high impact movements); and (3) minimizing activities that require bending/reaching down to the floor without assistance. These strategies were implemented with the goal of making the games more accessible and encouraging participants to work at their own levels of comfort and ability without disrupting the flow of the games and opportunities for team competition.

Second, we observed that there was a need to improve the game setup and instructions. Approximately 30 min is allotted for the group PA sessions. Thus, facilitators had a relatively short period of time to set up, explain, and implement the game each session. Several solutions were generated, including: (1) reviewing the instructions with participants in multiple formats, including asking participants to read the written instructions on the participant handouts, reviewing these instructions verbally, and providing a brief visual demonstration; and (2) providing facilitators with videos of the games to watch in advance and aid in explaining the games.

We have some initial evidence that these adaptations were successful, as we observed improvement on the item, “Participants understand how to complete the physical activity component (e.g., follow directions, able to complete after a practice round)” from Cohort 1 (*M* = 1.83, *SD* = 0.71) to Cohort 2 (*M* = 3.33, *SD* = 0.69), which was assessed as part of the systematic observation checklist.

### FitBit results

Results indicated that participants were compliant with wearing the FitBits and tracking their steps. On average participants had 6.66 ± 0.50 days of wear/week in Cohort 1 and 6.81 ± 0.30 days of wear/week in Cohort 2. Across the intervention period, average daily steps/week ranged from 8386.58 ± 3469.33 to 9368.71 ± 5936.19 in Cohort 1 and 7672.04 ± 4053.38 to 9371.40 ± 4591.66 in Cohort 2. Furthermore, we found that most participants successfully contributed to meeting the group-based goals, with individuals meeting the group-based goal an average of 6.56 ± 2.28 times in Cohort 1 and 6.78 ± 1.95 times in Cohort 2 (out of 9 possible sessions).

## Discussion

The present study described how formative process evaluation was used to monitor reach, dose, and fidelity, and provide timely, corrective feedback and solutions to implementation barriers during years one and two of the TEAM-PA randomized controlled trial. Results from Cohort 1–2 indicated relatively high levels of attendance, which were further enhanced through the use of makeup sessions. Facilitator dose delivered and fidelity to theoretical elements also exceeded implementation criteria, suggesting that intervention sessions were typically implemented as planned and facilitators were adhering to the theoretical essential elements. Fidelity at the group level was adequate, but somewhat lower than fidelity at the facilitator level. Furthermore, while most of the group-based PA games were implemented as planned, the process evaluation approach helped to identify several key areas for improvement. Results also indicated that most participants were compliant with wearing the FitBits, tracking their steps, and contributing to meeting the group-based goals. The integration of FitBit data to evaluate implementation of group-based goal-setting is a novel aspect of our process evaluation approach, as previous studies have primarily focused on individual-level adherence and acceptability [52, 53]. Taken together, the results from this study suggest that a comprehensive process evaluation framework that monitors fidelity at both the facilitator and group levels, integrates engagement data from wearables, and implementation of PA sessions, may help to improve implementation of complex, multi-theoretical frameworks in community-based settings.

A novel component of the TEAM-PA intervention includes a focus on intragroup competition, as past group-based interventions with African American women have typically focused on non-competitive forms of PA, such as walking or aerobics [12, 37, 38]. Results from this study revealed a few key areas for improvement regarding the competitive group-based PA games. One inherent challenge with group-based programs is the variability across group members, especially in terms of physical fitness and mobility. Thus, the use of formative process evaluation was particularly important to capture potential implementation barriers and generate timely solutions. Although most of the games were implemented as planned, the systematic observations revealed the need for additional modifications, including allowing for individuals to take breaks, incorporating a broader range of exercises, and minimizing activities that required bending/reaching down without assistance. We found that these strategies allowed participants to work at their own comfort level without disrupting the flow of the game and undermining the group competitive elements. Furthermore, we found that improving the game setup and instructions (e.g., by providing instructions in multiple formats and additional training videos for facilitators) was associated with a positive improvement in participants’ comprehension of the games from Cohort 1 and 2. The TEAM-PA process evaluation will continue to document strategies for improving implementation of group-based PA games, which may provide guidance for future group-based PA interventions.

The results from this study also revealed several strengths during the first two cohorts of this ongoing trial, including high levels of reach (e.g., attendance). Although there have been some improvements in recent years, attendance remains a key challenge among group-based PA interventions, including studies with African American women [12, 54, 55]. We used several strategies aimed at reducing barriers to participation, including limiting group sessions to one meeting per week and offering makeup sessions in the event of an absence. Makeup sessions have been found to be a useful strategy in past group-based interventions for enhancing dose and reach, while also offering opportunities for building positive relationships between participants and research staff [51]. Weekly reminder calls/texts, door prizes, and the availability of childcare were also used to encourage attendance, as in past trials [45, 51]. Additionally, we used strategies during the group orientations, which have been shown to improve attendance and retention, such as explaining the study rationale without scientific jargon and setting clear expectations about participation in the study, including attendance [56]. Past studies have also found that the use of group cohesion strategies is associated with higher attendance rates in group-based programs [57, 58], suggesting that the broader intervention approach (e.g., targeting social affilitation and collectivism) may be related to the high attendance rates.

This study assessed fidelity at both the facilitator and group levels. Adequate fidelity was reached at both levels, but values were somewhat lower at the group-level. Increasingly, process evaluation frameworks and guidelines are recognizing the importance of contextual factors for understanding differences across sites/groups [18, 59]. Although efforts are made to reinforce a positive group climate (e.g., via facilitator training, group ground rules) and hold certain group characteristics constant (e.g., group size, all female and African American participants), there is likely to be some variability between groups that may contribute to dynamics and relationships within groups [10]. The results from this study suggest that group-level fidelity is complex and warrants close monitoring through a systems-based process evaluation approach. Approaches for increasing group-level fidelity may include incorporating additional activities/strategies aimed at increasing group cohesion and using booster training sessions to reinforce facilitator training related to group-based behavioral skills and a positive group climate. Furthermore, pairing systematic observations with qualitative feedback from participants may provide a richer understanding of group-level fidelity and contextual factors related to group dynamics.

This study has some limitations. The results of the process evaluation were critical for assessing implementation strengths and areas of improvement within the TEAM-PA trial, but may not generalize to other interventions, given the relatively small number of intervention groups. While a comprehensive process evaluation framework was developed, future research is needed to evaluate how intervention implementation relates to the primary outcomes and mechanisms in the larger TEAM-PA trial. Despite these limitations, the present study provides an informative example of how formative process evaluation can be used during the early stages of a randomized controlled trial to monitor reach, dose, and fidelity, and provide timely, corrective feedback and solutions to implementation barriers.

## Conclusion

Findings from this study demonstrated high levels of reach, dose, and fidelity, while also highlighting strategies for implementing competitive group-based PA games that are accessible across physical fitness levels. We found that using formative process evaluation, including open-ended items, was important for capturing potential barriers to implementing competitive group-based PA games and that collaboratively brainstorming with our research staff and participants was an effective approach for generating timely solutions. Importantly, there was initial evidence that this formative process evaluation approach resulted in positive improvements in the implementation of the intervention across cohorts. Future group-based interventions for African American women may benefit from some of the adaptations identified in this study, including allowing for individuals to take breaks, incorporating a broader range of exercises, and minimizing activities that require bending/reaching down without assistance. In addition to using systematic observations and attendance tracking, future group-based interventions may also benefit from integrating data from wearables to evaluate whether participants are engaging in PA and goal-setting as intended. In summary, close monitoring of intervention implementation in community-based settings via formative process evaluation holds tremendous potential for improving intervention effectiveness, identifying important contextual variables, and increasing the likelihood of achieving meaningful changes in PA.

## Data Availability

Data will be available from the corresponding author on reasonable request after the trial is complete.

## Abbreviations

PA: Physical activity
SDT: Self-determination theory
SCT: Social cognitive theory
GDT: Group dynamics theory
